# Metabolic Roles of Plant Mitochondrial Carriers

**DOI:** 10.3390/biom10071013

**Published:** 2020-07-08

**Authors:** Alisdair R. Fernie, João Henrique F. Cavalcanti, Adriano Nunes-Nesi

**Affiliations:** 1 Max-Planck-Instiute of Molecular Plant Physiology, 14476 Postdam-Golm, Germany; 2 Instituto de Educação, Agricultura e Ambiente, Universidade Federal do Amazonas, Humaitá 69800-000, Amazonas, Brazil; jcavalcanti@ufam.edu.br; 3 Departamento de Biologia Vegetal, Universidade Federal de Viçosa, Viçosa 36570-900, Minas Gerais, Brazil

**Keywords:** amino acid, biological function, ion, inner mitochondrial membrane, mitochondrial carrier family, organic acid, substrate specificity, transport mechanism, vitamin

## Abstract

Mitochondrial carriers (MC) are a large family (MCF) of inner membrane transporters displaying diverse, yet often redundant, substrate specificities, as well as differing spatio-temporal patterns of expression; there are even increasing examples of non-mitochondrial subcellular localization. The number of these six trans-membrane domain proteins in sequenced plant genomes ranges from 39 to 141, rendering the size of plant families larger than that found in *Saccharomyces cerevisiae* and comparable with *Homo sapiens*. Indeed, comparison of plant MCs with those from these better characterized species has been highly informative. Here, we review the most recent comprehensive studies of plant MCFs, incorporating the torrent of genomic data emanating from next-generation sequencing techniques. As such we present a more current prediction of the substrate specificities of these carriers as well as review the continuing quest to biochemically characterize this feature of the carriers. Taken together, these data provide an important resource to guide direct genetic studies aimed at addressing the relevance of these vital carrier proteins.

## 1. Introduction

The acquisition of the mitochondrial endosymbiont brought a wide range of novel metabolic capabilities to the ancestral eukaryotic lineage [1]. Alongside efficient synthesis of ATP via the process of oxidative phosphorylation, the mitochondria are also the site of numerous other anabolic and catabolic pathways. The host cell has exploited this and depends on the mitochondria as a source of carbon skeletons for several further metabolic pathways including nitrogen assimilation, photorespiration, C_1_ metabolism, photosynthesis in C_4_ and crassulacean acid metabolism as well as the utilization of storage pools of carbon and nitrogen during seed germination [2,3]. Mitochondria additionally play roles in the biosynthesis of amino acids, tetrapyrroles, fatty acids and vitamin co-factors [4,5]. In order to achieve this, the mitochondrial matrix needs to be supplied by a wide range of solute transporters. Intriguingly, in a model of enzymes allocated to specific cellular compartments of Arabidopsis, Mintz-Oron et al. [6] revealed that approximately half of the reactions could be assigned to specific subcellular compartments based on experimental evidence. For the remainder, they predicted the most likely subcellular location based on a parsimony principle of minimizing the number of intracellular transporters required to activate the reactions with a known localization in the corresponding compartments [6]. This method predicted that a metabolic network of some 1200 reactions (compartmented among the cytosol, plastid, mitochondrion, endoplasmic reticulum, peroxisome, vacuole and Golgi apparatus) required a phenomenal 772 intracellular transporters. Similarly, at least 228 metabolites and 89 transport processes are required in the minimal human mitochondrial metabolic network [7], suggesting that the total number of solute transporters currently catalogued to reside in the plant mitochondria may be insufficient to account for all transport steps required [8]. It is, however, important to note that this list contains not only MCF (mitochondrial carrier family) members but also members of other families [8]. Although the outer mitochondrial membrane is permeable to small solutes (with a molecular mass of less than 4–5 Da) [9,10,11], the inner membrane is impermeable with only very small uncharged molecules such as O_2_ and CO_2_ able to readily pass through this membrane. The passage of hydrophilic compounds across the inner mitochondrial membrane is mainly catalyzed by the nuclear encoded mitochondrial carrier family (MCF) [12,13,14,15]. MCs (mitochondrial carriers) are small proteins ranging in size from 30–34 kDa and possess common defining structural features. Their primary structure is characterized by three tandemly repeated, approximately 100 amino acid long, homologous domains with each repeat containing two hydrophobic segments, which span the membrane, and a characteristic amino acid sequence motif PX[D/E]XX[K/R]/RX[K/R] (20–30 residues) [D/E]GXXXX[W/Y/F][K/R]G (PROSITE PS50920, PFAM PF00153 and IPR00193). Two sub-families, the aspartate/glutamate and ATPMg-Pi carriers, have additional N-terminal regulatory domains of approximately 150 amino acids that usually contain Ca^2+^-binding motifs [12,16]. The molecules transported by the MCF are highly variable in size and structure, ranging from H^+^ and NAD^+^ and coenzyme A. They also display a range of ionic charges being either positive, negative or zwitterionic at physiological pH. They often act as antiporters, although uniport transport and H^+^-compensated anion symport is also mediated by some MCs. Furthermore, MCs can be subdivided on the basis of their electrical nature with for example the ADP/ATP and aspartate/glutamate transporters drive electrogenic reactions (which result in net charge transfer) whereas the carrier subfamilies for phosphate (Pi), glutamate, and GTP/GDP as well as for 2-oxoglutarate and ornithine are electroneutral.

Considerable research has been conducted on characterizing members of the MCF in both yeast and animals (see [13,14,17,18,19,20,21,22,23,24,25,26,27] for reviews). Regarding other eukaryotes, the MCF members of the early-branching kinetoplastid parasite *Trypanosoma brucei* have been studied by sequence and phylogenetic analyses [27]. This study gave new insights into the evolution and conservation of the 24 identified MCF homologues identified in that organism [27]. In recent years the advent and exploitation of systems biology approaches have provided considerable insight into the putative in vivo function of plant MCFs, whilst the adoption of recombinant enzyme approaches have allowed the biochemical characterization of their functions. In this article, we summarize the structure and transport mechanisms of members of the MCF family, discuss its expansion in plants and finally summarize the biochemical characterization of the transport properties of MCF members that have been reconstituted in liposomes. The reader is referred to our other article in this issue [28] for information concerning the, sometimes unusual, subcellular localization of these proteins and the characterization of transgenic loss-of-function lines.

## 2. Structure and Transport Mechanisms

**Figure 1:**
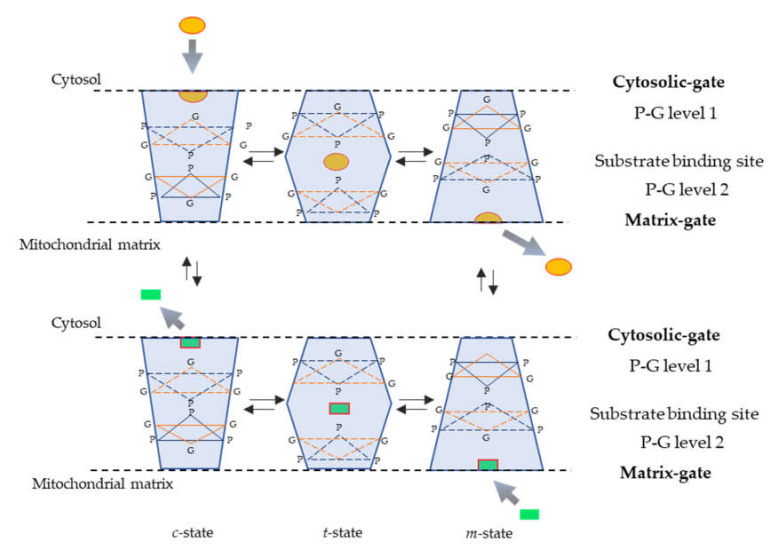
Mechanism of substrate translocation catalyzed by mitochondrial carriers. Simplified scheme depicting the transition of mitochondrial carriers from the *c*-state to the *m*-state and vice versa as previously proposed [14]. The trapezoid shape on the left is used to illustrate the *c*-state after the release of the substrate towards the cytosol and immediately after the entry of the substrate from the cytosolic side; the trapezoid shape on the right illustrates the *m*-state after the release of the substrate into the matrix and immediately after the entry of the substrate from the matrix side; and the two central hexagonal shape solids depict the transition states (*t*-state) of the carrier with the bound substrate entered from the cytosol and from the matrix. The yellow disk and green rectangle shapes represent the substrates entering from the cytosol and from the matrix, respectively; orange triangles represent closed gates, and dotted orange triangles indicate open or partially closed gates. All transport steps are fully reversible. The positions of the salt bridge networks (cytosolic and matrix gates), P-G level 1, substrate binding site and P-G level 2 are indicated on the right.

Due to its high abundance, the ADP/ATP carrier (AAC) is the member of the family that has been studied the most. It is the first mitochondrial carrier for which a high-resolution X-ray structure was provided [29]. The bovine carrier was crystallized in the presence of a strong inhibitor, the carboxyatractyloside (CATR). The structure gives an insight not only into the overall fold of mitochondrial carriers in general but also into atomic details of the AAC in a conformation that is open toward the intermembrane space (IMS). The three dimensional structure of the ADP/ATP carrier is critical to our understanding in several other ways. First, it exhibits a three-fold pseudo-symmetry in lines with the three-fold sequence repeats mentioned above [30], similar to that observed by electron microscopy [31]. Secondly, it approximately corresponds to the *c* (cytosolic)-state of the ADP/ATP carrier since CATR blocks the carrier in this state [19]. Thirdly, this structure has become a much used template for the building of homology models of various carriers, greatly improving our understanding of MC structure/function relationships [32,33,34,35,36,37,38]. The structural fold observed in the bovine transporter was subsequently confirmed by the structures of two yeast isoforms of the ADP/ATP carrier. Intriguingly, the odd-numbered transmembrane alpha-helices have pronounced kinks at the Pro residue of the highly conserved signature motif Px[DE]xx[KR], with the P kink giving them a pronounced L shape which helps to block access of the central cavity from the mitochondrial matrix in the *c*-state (Figure 1). The charged residues of the signature motifs from inter-domain salt bridges [29,39] are now known as the matrix salt bridge network. Furthermore, residues of the salt bridge interact with a proximal glutamine residue which hydrogen bonds to both salt bridge residues forming a glutamine brace (Q brace [39]). These residues are highly conserved with one to three Q braces typically found in the SLC25 subfamily of the MCF. CATR inhibits the ADP/ATP carrier by binding tightly in the central cavity thus prevent translocation across the membrane. Just last year, the first structure of a mitochondrial carrier in the *m*-state has been solved—the ADP/ATP carrier from the thermotolerant fungus *Thermothelomyces themophilia* inhibited by bongkrekic acid [40]. The *m*-state structure displays the same three-domain architecture, but with the domains rotated compared with the *c*-state, opening the central cavity to the mitochondrial matrix and closing it to the intermembrane space and thereby disrupting the matrix network and Q brace [14]. On the intermembrane side the transmembrane helices are positioned closely together and form the interdomain cytoplasmic salt bridge network (Figure 1). This network is stabilized by hydrogen bonds with the hydroxyl bonds with the hydroxyl groups of the tyrosines of the motif forming a tyrosine brace (Y brace; [39]). Most SLC members have one to three Y braces. Having structural information for both *c*- and *m*-states has significantly advanced our understanding of how these proteins operate at the molecular level. These advances have been excellently covered elsewhere [25], so we will not detail them here except to say that the structural features are likely to be conserved, with few exceptions, throughout the MCF.

## 3. Extension of the MCF

Although only six MC proteins were sequenced following their purification from mitochondria or by DNA sequencing (see [41] and references therein) the genomic era has massively expanded our inventories of the MCFs of various species with *S. cerevisiae* encoding 35 [42], the human genome 50 [18] and *Arabidopsis thaliana* 58 [5]. The first step in identifying MC function is to search for the substrates transported by a specific carrier. In order to do so, the primary tools in our arsenal are phylogenetic clustering, genetic information, knowledge of cellular metabolism and complementation of phenotypes. However, such methods remain inconclusive and overly speculative. To date, the most effective strategy has been heterologous expression in *Escherichia coli* (see for example [43]) or *S. cerevisiae* (see for example [44,45,46]) and reconstitution of the subsequently purified recombinant carriers into liposomes in which direct transport assays are performed. To date, such gene-function studies have been carried out on 32, 40 and 26 of the MCFs of *S. cerevisiae*, human and *A. thaliana*, respectively. Focusing on the green lineage alone, MCs are highly abundant in the genomes of several species of dicots, monocots and algae with a 2020 update (Table 1 and Table S1) suggesting they range in number from 39 to 141, surpassing the 37 to 125 range when we last reviewed this family in 2011 [12]. In turn, a reasonable understanding of the apparently increasing number of predicted MCF genes in green line in comparison with the report of 2011 [12] is the development of powerful tools concerning both next generation sequencing approaches and/or bioinformatics algorithms for annotation and assembly of new, more accurate *de novo* reference genomes. In this review we demonstrate how the function of *A. thaliana* proteins could be reasonably, yet not completely, accurately predicted via examination of their symmetry-related triplets and subsequent comparison to those of MC subfamilies for which substrate specificities were determined in human, yeast or Arabidopsis itself. Those instances in which poor accordance was found between the prediction and experimental results can be split in two: those displaying novel substrate specificity and those residing at different subcellular localization [28]. Table 2 lists the main subfamilies that MCs can be partitioned into on the basis of their substrate specificities. It is, however, important to note that the caveats which we previously mentioned [12] remain valid. In brief: (i) some substrates are transported by more than one subfamily; (ii) the best transported substrate in reconstituted liposomes may not reflect the most important substrate under physiological conditions; (iii) some subfamilies may additionally transport as yet untested substrates (see for example [47]); and (iv) most of the subfamilies presented in this table are present in all eukaryotes. It has been suggested that key amino acids residues important for the transport mechanism are likely symmetrical, and those involved in substrate binding are likely asymmetrical (indicating the asymmetry of the substrates) [32,33,34,35,36,37,38]. Hence, scoring the symmetry of residues in the sequence repeats, it is possible to associate the substrate-binding sites and salt bridge networks that are important for the transport mechanism in family members [32,33,34,35,36,37,38]. Thus, the substrate specificity defined carrier subfamilies are also characterized by specific amino acid triplets with the number of characterizing triplets ranging from two to eight. Moreover, related subfamilies sharing some triplets, for example the NAD^+^, PyC and FAD families, share triplet 19 as well as some transport substrates [48,49] whereas the OGC and DTC subfamilies also share two triplets (KLK and GTY) as well as some transported substrates [43,50] substrate specificity. Finally, as in our previous study, the uncoupling protein (UCP) and unnamed transporters have been added despite the fact that the substrates are unknown for the latter. However, in contrast to our previous study [12], as detailed below, the substrate specificities of UCP have recently been characterized [51].

An additional method for analyzing function has been the deduction from phylogenetic trees—an approach that has also been used to address the evolution of the MCFs [12,52]. Intriguingly, either if all MCF members of a single representative of a kingdom [12] or multiple representatives of each kingdom but only a subset of the MCFs are used [52] similar broad conclusions can be made. These are namely that the MCFs are highly divergent, yet that the fact that the vast majority are conserved across plants, animals and yeast lineages suggests that many MC functions existed before the speciation events that have produced the three kingdoms [12]. Interestingly, however, the comparison of intraspecific paralogs suggests that these originated by gene duplication events that occurred independently in those three lineages [12]. At a finer level the comparison of tricarboxylic acid (TCA) cycle relevant MCFs alone revealed that mitochondrial organic acid transporters formed two distinct clades. In the first clade, dicarboxylate carriers (DICs) and dicarboxylate-tricarboxylate carrier (DTC) grouped with 2-OG carriers (OGCs). The succinate-fumarate carrier (SFC) formed the second organic acid clade with other non-plant organic acid transporters including oxodicarboxylate carriers (ODCs), citrate carriers (CiCs), and yeast suppressor of HM (histone-like proteins in yeast mitochondria) mutant 2 (YHM2). Biochemical data would indicate that DTC and CiC must be closely related as they both transport citrate; phylogenetic analysis revealed that SFC, and not DTC, is more similar to CiC [52]. Based on available biochemical data, it thus appears that the transport functions of CiC and DTC have evolved independently but perhaps convergently. As would be expected, the possibility to use phylogeny to detect orthologs between plants is much greater than across kingdoms [12]. The use of tools such as Orthofinder, which provides phylogenetic inference of orthologs [53], and PlaNet and FamNet, which include co-expression data to refine such searches [54,55], render such searches easier and will likely prove highly informative in improving our understanding of plant MCFs beyond Arabidopsis.

Further evolutionary insight into plant MCF members was attained by studying the location of introns in MC genes and examining the synteny between MCF members in the dicot *A. thaliana*, the monocot *Brachipodium distachyon* and the algae *Osterococcus luminarius* [12]. The first of these strategies took its cue from the observation that introns tend to interrupt the coding sequence of the human citrate, carnitine and dicarboxylate carrier genes at positions corresponding to or in the close vicinity of the hydrophilic loops in the MC amino acid sequences [56,57,58]. Comparison of all 58 members of the *A. thaliana* MCF revealed that hydrophobic loops host a notable excess in intron density compared with transmembrane helices, suggesting that introns are unrepresented in transmembrane helices due to negative selection [12]. In the second analysis, the co-linearity of regions of the *A. thaliana*, *B. distachyon* and *O. luminarius* genomes were exploited. In *O. luminarius* six MCF genes were found in co-linear regions of chromosomes 13 and 21, consistent with the known origin of chromosome 21 in this species [59]. In spite of several whole genome duplication events (see [60,61] for reviews) the same number of gene pairs were found in Arabidopsis, a fact best explained by high rate of gene loss and gene rearrangement in this species [62,63]. By contrast, the genome of *B. distachyon* contains six pairs of MC paralogs in co-linear segments, likely reflecting a lower rate of gene loss and gene rearrangement in this species. Fascinatingly, however, even though approximately 500 million years separate monocots and dicots from the common ancestor of the angiosperm, 15 MCs in Arabidopsis and 13 in Brachipodium are present in conserved synteny blocks with an over-representation for nucleotide carriers being apparent which has been suggested to reflect either that their preferential expansion is tolerated in angiosperms or, more likely, that they functionally contributed to angiosperm evolution [12].

## 4. Biochemical Characterization of Plant MCF Members

Out of 58 MCF members found in Arabidopsis genome, 17 genes have not been fully characterized and therefore the biochemical role of these proteins remains unknown. In the last 10 years, the biochemical functions of 21 MCs from Arabidopsis have been investigated (Table 2) and studies on the characterization of the physiological importance of these carriers in plants have been reported [28].

### 4.1. Coenzyme A Carriers

From the subfamily of nucleotides and dinucleotides carriers, two genes encoding for MCF proteins, At1g14560 and At4g26180, based on the presence of sequence motifs (symmetry-related amino acid triplets [12]) were described as potential coenzyme A (CoA) carriers [12]. Comparative genomic analysis allowed the identification of two homologs of these proteins in maize (*Zea mays*; GRMZM2G161299 and GRMZM2G420119) [64]. It was verified that all these proteins from maize and Arabidopsis are targeted to mitochondria and are also able to complement the growth wild type phenotype in the yeast leu5D mutant [65] defective for the mitochondrial CoA carrier [64]. These proteins also restored the mitochondrial CoA level in the same yeast mutant. These results clearly demonstrated that these proteins catalyze the transport of CoA through the mitochondrial membrane. It is noteworthy that, to our knowledge, the substrate specificity of this transporter has not yet been fully investigated. This is particularly important, because in addition to CoA, these transporters might also have capacity to transport other substrate or substrates. In this regard, it was reported that the Arabidopsis peroxisomal NAD carrier PXN, in addition to NAD^+^, NADH, AMP, ADP and adenosine 3’, 5’-phosphate (PAP), is also able to catalyze CoA transport [66]. PXN is encoded in Arabidopsis by the gene At2g39970 and was investigated regarding its substrate specificity and the transport properties by using a wide range of potential substrates [66]. Detailed biochemical analyses demonstrated that PXN catalyzes fast counter-exchange of substrates and much slower uniport [66]. In the same study, it was shown that the transport catalyzed by PXN is saturable with a submillimolar affinity for NAD^+^, CoA and other substrates. More recently, the physiological function of PXN in plants was further investigated [67]. Interestingly, by using *S. cerevisiae*, uptake analyses indicated that PXN has a low affinity for CoA, which suggests that the PXN function of the CoA transporter might not be possible under physiological conditions. Complementing diverse mutant yeast strains with PXN and investigating the suppression of the mutant phenotypes, the authors provided evidence that PXN is not able to function as a CoA transporter or a redox shuttle by mediating a NAD^+^/NADH exchange, but instead catalyzes the import of NAD into peroxisomes against AMP in intact yeast cells [67]. This work demonstrated that Arabidopsis PXN supplies the peroxisomes with NAD by importing this coenzyme from the cytosol in exchange with AMP.

### 4.2. Nicotinamide Adenine Dinucleotide (NAD) Carriers

Regarding NAD transport in mitochondria and plastids, in addition to PXN, it has been demonstrated that two MCF members in Arabidopsis, named *At*NDT1 and *At*NDT2, are able to catalyze the import of NAD in these organelles [68]. Both carriers are able to complement the phenotype of a yeast mutant lacking NAD^+^ transport [68]. Surprisingly, both *At*NDT1 and *At*NDT2 exhibit similar substrate specificity, being able to import NAD^+^ against ADP or AMP, and not accepting NADH, NADP^+^, NADPH, nicotinamide or nicotinic acid as transport substrates [68]. Intriguingly, despite the similarities in terms of biochemical properties, initial localization analysis indicated that *At*NDT1 was located in the plastid membrane while *At*NDT2 was in the mitochondrial membrane [68]. Surprisingly, *At*NDT1 was found in mitochondrial membranes in proteome studies [69] and previously a GFP-tagging and immunolocalization study was not able to find *At*NDT1 targeted to chloroplast membranes [70]. Very recently, both *At*NDT1- and *At*NDT2-GFP fusion proteins were found exclusively located in the mitochondria, clearly indicating their mitochondrial localization [71].

### 4.3. Adenylate Carriers

The transport catalyzed by the ADP/ATP carrier plays an important role in sustaining the cellular ATP homeostasis by facilitating the counter exchange of mitochondrial ATP for cytosolic ADP [72]. ADP/ATP carrier proteins have been identified and characterized in different species including organisms of medical and veterinary importance, such as *T. brucei* [27,73,74]. The importance of the efficient adenylate transport systems for intracellular energy partitioning between the cell organelles has been widely demonstrated in plants (for review see [72]). Adenylate carriers found in different organelles have been previously identified and biochemically characterized in plants. There are three subgroups of MCF responsible for adenylate transport in plants: (1) the well-characterized ADP/ATP carriers, named AAC carriers (*At*AAC1, At3g08580; *At*AAC2, At5g13490; and *At*AAC3, At4g28390), which are required for mitochondrial energy passage (for review see [72]) and represent the most abundant proteins in the inner mitochondrial membrane (*At*AAC1–3; 53,065 protein copies/mitochondria [75]); (2) the mitochondrial ATP-Mg/phosphate carriers, named as APC carriers (*At*APC1, At5g61810; *At*APC2, At5g51050; and *At*APC3, At5g07320); and (3) the adenine nucleotide transporter ADNT1 (At4g01100), which transport AMP instead of ADP as counter exchange substrate of ATP [76].

In Arabidopsis there are three genes encoding putative APC proteins (*At*APC1–3). These proteins belong to the MCF and exhibit high amino acid sequence similarities to their human and yeast counterparts [12,19]. It was demonstrated that all APC proteins from Arabidopsis localize to mitochondria and restore the growth phenotype of APC yeast loss-of-function mutants [77]. Interestingly, these carriers interact with calcium (Ca^2+^) via their N-terminal EF-hand motifs *in vitro*, suggesting that APC1–3 isoforms represent Ca^2+^-regulated ATP-Mg/phosphate transporters. Insights into the biochemical characteristics of these APCs were reported based on reconstitution of heterologously expressed proteins into liposomes [16,78]. The obtained results demonstrated that Arabidopsis APCs mediate antiport of ATP, ADP and phosphate and the transport characteristics indicated that the plant APCs preferentially import the Ca^2+^- and not the Mg^2+^-complexed form of ATP, at least in an *in vitro* system [78]. It is important to note that recent evidence indicates that not only Mg^2+^ and Ca^2+^, but also other divalent cations and specifically Mn^2+^, Fe^2+^, Zn^2+^ and Cu^2+^, are transported together with ATP by human and Arabidopsis APCs [79].

### 4.4. Amino Acid Carriers

Another enigmatic transporter named A BOUT DE SOUFFLE (BOU) was identified in Arabidopsis At5g46800 a long time ago [117]. Previous studies extensively characterized the physiological function of the BOU transporter in plants and revealed that this protein plays important roles related to fatty acid β-oxidation [117], photorespiration and growth of meristem cells [118]. However, the specific substrate for the BOU transporter protein was unknown until recently [104]. Detailed biochemical characterization of Arabidopsis BOU and Ymc2p, the BOU homolog from *S. cerevisiae*, revealed the transport properties and kinetic parameters of these proteins. Both Ymc2p and BOU proteins are able to transport glutamate, and to a lesser extent L-homocysteinesulfinate, but no other amino acids nor many other tested metabolites [104]. This study also revealed that both proteins Ymc2p and BOU catalyze unidirectional transport of glutamate and, as reported for other known MCs, a faster counter exchange mode of transport, and catalyze a transmembrane glutamate^−^ + H^+^ symport. These results led to the conclusion that for both Ymc2p and BOU, the physiological function of these proteins is to catalyze the import uptake of glutamate into the mitochondria.

In Arabidopsis, two MCs (*At*BAC1, At2g33820; and *At*BAC2, At1g79900) are able to transport basic amino acids [106,119,120]. *At*BAC1 shares a 36% identity with BOU, whereas *At*BAC2 is 40% similar to SLC25A29, although it is also related to BOU (36% identity) and aspartate/glutamate carriers (AGCs, 30–33% identity) [22]. Recombinant proteins from *At*BAC1 and *At*BAC2 were purified and reconstituted in liposomes [106,120]. The results indicated that both proteins transport lysine, arginine, ornithine and histidine [106,120]. Interestingly, it was verified that only *At*BAC2 transports the neutral amino acid citrulline [106,120]. In addition, these studies indicated that *At*BAC1 and *At*BAC2 exhibit differences in terms of substrate specificity, with *At*BAC2 being less specific for L-amino acids. Despite the similar biochemical properties, the physiological roles of *At*BAC1 and *At*BAC2 seem to be different. While *At*BAC1 is likely involved in remobilization of storage compounds after seed germination in Arabidopsis and rice [106,119,121], *At*BAC2 is more related with stress responses being expressed especially in responses to hyperosmotic stress and also during senescence [106,119,122,123].

### 4.5. Uncoupling Proteins

Uncoupling proteins (UCPs) have been described as being involved in dissipation of proton gradients across the inner mitochondrial membrane that is normally used for ATP synthesis [92,93,94,95,96,97,98,99,100,101,102,103,104,105,106,107,108,109,110,111,112,113,114,115,116,117,118,119,120,121,122,123,124]. Homology analysis with UCP from humans revealed that six genes in the Arabidopsis genome (*At*UCP1–6) encode putative UCPs [124,125,126]. It was previously demonstrated that the isoform *At*UCP1 (At3g54110) is localized to mitochondria and exhibits the activity of an uncoupling protein similar to the human UCP1 [124,125,126]. The function of the isoform *At*UCP2 (At5g58970) was less understood until recently because it was detected in the Golgi apparatus [127] and also in the plasma membrane [128]. Recently, it was shown that *At*UCP2 isoform is also a mitochondrial localized protein [51]. Intriguingly, both isoforms *At*UCP1 and *At*UCP2 were shown to transport amino acids (glutamate, aspartate, cysteine sulfinate, and cysteate), dicarboxylates (malate, oxaloacetate, and 2-oxoglutarate), phosphate, sulfate, and thiosulfate [51]. Further biochemical analyses revealed that both isoforms catalyze an electroneutral aspartate/glutamate heteroexchange activity, in contrast to that mediated by the mammalian mitochondrial aspartate glutamate carrier. Three other former members of the *At*UCP subfamily of Arabidopsis MCF (*At*UCP4–6) were renamed as dicarboxylate carriers (DIC) (*At*DIC1, At2g22500, *At*DIC2, At4g24570; and *At*DIC3, At5g09470) since these proteins are able to transport oxaloacetate, malate, succinate, phosphate, sulfate, thiosulfate and sulfite [93].

### 4.6. Dicarboxylate Carriers

As mentioned above, in the Arabidopsis genome three potential homologues of yeast and mammalian mitochondrial DICs were found and designated as *At*DIC1–3 (AT5G09470) [93]. *At*DIC3 shares only 55–60% identical amino acids with *At*DIC1 and *At*DIC2, whereas *At*DIC1 and *At*DIC2 share 70% identical amino acids, suggesting that *At*DIC1 and *At*DIC2 are more closely related [93]. Interestingly, a recent Arabidopsis mitochondrial proteomic study verified that *At*DIC3 is not highly expressed in comparison with *At*DIC1–2, as *At*DIC1 is more abundant than *At*DIC2 (59 and 21 protein copies per mitochondria respectively) [75]. Transport experiments with recombinant and reconstituted *At*DIC proteins demonstrated that the substrate specificity of these proteins is unique to plants, indicating the combined characteristics of the DIC and oxaloacetate carrier in yeast [93]. Indeed, the Arabidopsis DICs transport a wide range of dicarboxylates including malate, oxaloacetate and succinate as well as phosphate, sulfate and thiosulfate at high rates, whereas 2-oxoglutarate was revealed to be a very poor substrate. In the same study, the kinetic properties of recombinant *At*DIC1–3 proteins were determined [93]. It was shown that for all *At*DIC proteins, Vmax is not significantly different for the three substrates tested (malate, sulfate and phosphate). Nevertheless, the Vmax for *At*DIC3 was higher than the values observed for *At*DIC1 and *At*DIC2. Regarding the transport affinity (Km) of *At*DIC1–3 proteins, for sulfate it was lower than the Km values for phosphate and malate. For *At*DIC3, it was verified that the Km for sulfate was one order of magnitude lower than the Km values for malate and phosphate; furthermore, the Km of *At*DIC3 for sulfate was 3–4-fold lower than the Km values of *At*DIC1 and *At*DIC2 using the same substrate. The identification and characterization of the biochemical properties of DIC proteins in Arabidopsis led to different questions about the physiological roles of these carriers in plants under distinct physiological conditions. Surprisingly, according to our current knowledge, the isolation and characterization of mutant plants for each *At*DIC isoform still need to be performed.

### 4.7. Dicarboxylate/Tricarboxylate Carrier

Dicarboxylate/Tricarboxylates carriers (DTCs) are mitochondrial transporters that are able to transport both dicarboxylic acids (such as malate, maleate, oxaloacetate and 2-oxoglutarate) and tricarboxylic acids (such as citrate, isocitrate, *cis*-aconitate and *trans*-aconitate) [50]. In the human parasite *Trypanosoma brucei*, it was demonstrated that a plant-like mitochondrial carrier family protein, named *Tb*MCP12, is able to transport both dicarboxylates and tricarboxylates across the inner mitochondrial membrane (IMM) [129]. Silencing this carrier in *T. brucei* was not lethal, while its overexpression was deleterious. These results indicated that the intracellular abundance of *Tb*MCP12 is involved in the regulation of NADPH balance and mitochondrial ATP-production. In plants, it was recently demonstrated that DTCs are the most abundant mitochondrial carrier proteins in the IMM of Arabidopsis, comprising 0.8% of the total IMM area (6836 protein copies per mitochondria) [75]. Interestingly, unlike the other three more abundant carrier proteins in the IMM, i.e., ADP/ATP carriers (*At*AAC1–3; 53,065 protein copies/mitochondria), mitochondrial phosphate carriers (*At*MPT2–3; 21,325 protein copies/mitochondria) and uncoupling proteins (*At*UCP1–3; 8595 protein copies/mitochondria), only one DTC homolog is found in Arabidopsis (At5g19760). In addition to Arabidopsis, DTCs have been described in several plant species including tobacco (*Nicotiana tabacum*) [50], grapes (*Vitis vinifera*) [130] citrus (*Citrus junos*) [131], Jerusalem artichoke (*Helianthus tuberosus*) [132] (and maize (*Zea mays*)) [133]. Surprisingly, the numbers of DTC homologs found in different plant species vary without a clear pattern, for example, in the Brassica genus, the number of DTC homologs varies from one in *A. thaliana* and *Arabidopsis lyrata*, two in *Brassica oleracea*, and three in *Brassica rapa* [52]. In tobacco, four homologs (*Nt*DTC1–4) were identified [50].

For *At*DTC and *Nt*DTCs, the transport activity involves an obligatory electroneutral exchange of dicarboxylates such as malate and 2-oxoglutarate and tricarboxylates such as citrate [50]. In addition to catalyzing the dicarboxylate/tricarboxylate transport activity, it has been demonstrated that DTCs are able to catalyze homoexchange transport activities, such as dicarboxylate/dicarboxylate and tricarboxylate/tricarboxylate [50]. It is unclear so far which of these modalities are relevant in in vivo plant systems. From *in vitro* transport assays it is possible to conclude that DTCs are promiscuous in terms of transported substrates [50]. In the same study, it was observed that the highest DTC activities are in the presence of internal 2-oxoglutarate, malate, maleate, oxaloacetate, succinate or malonate. Intriguingly, it was observed also that citrate, isocitrate, *cis*-aconitate, *trans*-aconitate, and sulfate were exchanged for external 2-oxoglutarate, although to a slightly lower extent than the dicarboxylates [50]. Any significant exchange was observed using internal fumarate, phosphoenolpyruvate, phosphate, pyruvate, glutamate, aspartate, glutamine, carnitine, ornithine, or ADP [50]. Together, these results demonstrated that DTCs are able to transport several intermediates of the TCA cycle, with the exception of succinyl-CoA and fumarate for which there is no available information. Another interesting characteristic of DTCs is the pH dependence. It was demonstrated that DTC-mediated oxoglutarate and citrate homoexchanges were dependent on pH, as the oxoglutarate/oxoglutarate and citrate/citrate exchanges increased on decreasing the pH from 8.0 to 5.5 for both *Nt*DTC1 and *At*DTC. For *At*DTC, the homoexchange kinetic constants measured for different substrates in two different pH values indicated that regardless of the substrate, the Km and Vmax varies as a function of pH value. Interestingly, the Km values were increased at pH 7, suggesting that the substrate affinities were reduced; Vmax values were also decreased at pH 7. Of note, the modulation of transport kinetics by pH is highly important for plant metabolism because it has been demonstrated for Arabidopsis that in the mitochondrial matrix the pH is around 8.1 and that in the cytosol the pH is close to 7.3 [134].

### 4.8. Succinate/Fumarate Carriers

In Arabidopsis, one of the MCF members (At5g01340), named as SFC1 carrier, exhibits 35% similarity with the ACR1 transporter from yeast [96]. The yeast SFC1 is able to transport fumarate, succinate, methylfumarate, 2-OG and OAA against [^14^C]oxoglutarate [96]. The SFC1 transporter was further shown to prefer succinate and fumarate as substrates since the presence of either substrate almost completely inhibits fumarate/[^14^C]oxoglutarate exchange [96]. The Arabidopsis SFC1 homolog complemented the *arc1* yeast mutant re-establishing the yeast growth in minimal media with ethanol as the sole carbon source [95]. Despite the predictions and preliminary biochemical information in plants, the biochemical evidence in favor of succinate/fumarate transport is still lacking. Moreover, recently the SFC1 sequence from Arabidopsis was expressed in *E. coli* and protein was purified and reconstituted in liposomes [94]. Surprisingly, the results of transport properties and kinetic parameters revealed that *At*SFC1 transports mainly citrate, isocitrate and aconitate and, to a lesser extent, succinate and fumarate. Furthermore, it was demonstrated that the *At*SFC1 carrier catalyzes a fast counter-exchange transport and low uniport of substrates, as well as exhibiting a higher transport affinity for tricarboxylates than dicarboxylates [94]. Intriguingly, there have been both reports and model predictions in Arabidopsis showing net influx of succinate to the mitochondria, which would have been expected as succinate is the preferred substrate of non-plant SFCs. Thus, it is likely that another unidentified transporter is using succinate as a counter-substrate to facilitate fumarate transport.

### 4.9. Phosphate Carriers

Apart from ADP, the transport of phosphate (Pi) through the IMM is essential for the oxidative phosphorylation of ADP to ATP. In Arabidopsis, three genes encodes mitochondrial Pi carriers, namely *At*MPT1 (or PiC3; At2g17270), *At*MPT3 (or PiC1; At5g14040) and *At*MPT2 (or PiC2; At3g48850), and all of them are related to mitochondrial Pi carrier (PiC) from human and yeast [12,105]. Biochemical studies demonstrate that Arabidopsis PiC1 and PiC2 complement yeast mutants deficient in mitochondrial Pi import [106,107], thus confirming that these proteins act as PiCs. Surprisingly, the role of the Arabidopsis PiC3, which is more distantly related to the other PiC1 and 2 plant isoforms [12,135] remains to be elucidated. Interestingly, ADP/ATP carriers (*At*AAC1–3; 53,065 protein copies/mitochondria) and PiC1–2 (or *At*MPT2–3; 21,325 protein copies/mitochondria) are the most abundant proteins in the IMM [75]. In agreement, it has been proposed that PiCs in the inner mitochondrial membrane are able to physiologically interact with AAC transporters, catalyzing a Pi/H^+^ symport (or Pi/OH^−^ antiport) and thus supplying phosphate required for the ATP synthesis [136,137]. Recently, it was shown in Arabidopsis that a putative Pi transporter interacts with TCA cycle enzymes [138,139]. Notwithstanding, the significance of these protein–protein interactions at physiological levels remains to be elucidated.

### 4.10. Pyruvate Carriers

Pyruvate, the final product of glycolysis in the cytosol, must be transported into mitochondria to supply the carbon skeletons for oxidative metabolism through the TCA cycle reactions. The transport of pyruvate through the IMM must be performed by specific carriers. While candidates for mitochondrial pyruvate carriers (MPCs) have not been identified in the classic MCF yet, the identity and functionality of a series of MPCs, non-MCF members, have been reported in yeast, *T. brucei*, drosophila, mouse and humans [140,141,142]. The biochemical properties of MPCs have been extensively studied and expertly reviewed [143,144,145,146] mainly due to the research efforts to understand the importance of MPCs in metabolism-related human diseases. Furthermore, in *S. cerevisiae* it was demonstrated that MPC is a hetero-dimer in its functional state providing the basis for the structure elucidation of the functional complex [147]. In plants, the biological functions and molecular mechanisms involving MPCs are not well understood. Bioinformatics analysis suggests that a protein named NRGA1, a negative regulator of guard cell abscisic acid (ABA) signaling (At4G05590), shares homology with the MPC2 proteins from yeast, drosophila, human and mouse [148]. Besides NRGA1 protein, four other MPC candidates are encoded by the Arabidopsis genome [149]. This family of MPCs from Arabidopsis are phylogenetically classified into three categories: MPC1 (At5G20090), MPC2-like proteins (At4G14695, At4G22310 and At4G05590) and At4G26780 [149]; that said, little is known regarding the functions of these proteins. So far it is known that NRGA1 is located in the mitochondria and its sequence exhibits transmembrane domains [148]. Furthermore, in Arabidopsis this putative MCP2-like protein seems to be involved in stomata ABA signaling [148]. Interestingly, a recent study demonstrated that *At*MPC1 interacts with NRGA1 and plays a role in the regulation of stomatal movement and pyruvate cellular content [150]. In addition, it was demonstrated with yeast MPCs that by mimicking the physiological pH gradient between the mitochondria and the cytosol, a quantifiable pyruvate transport was observed, whilst in the absence of the pH gradient no transport of pyruvate was observed [147]. Recently, it was demonstrated that the formation of *At*MPC protein complexes is required for cadmium (Cd) tolerance and also prevention of Cd accumulation in Arabidopsis [151]. In the same study, it was demonstrated that *At*MPC complexes are composed of two elements, the *At*MPC1 and *At*MPC2 (*At*NRGA1 or *At*MPC3). Interrupting the formation of *At*MPCs by silencing *At*MPC1 element, the synthesis of acetyl-coenzyme A was supplemented by glutamate and thus sustaining the activity of TCA cycle reactions and glutathione synthesis following exposure to Cd stress [151]. Clearly, more molecular, biochemical and physiological research efforts are still needed to understand the transport mechanism, substrate specificities and physiological roles of mitochondrial pyruvate transporters in plants.

### 4.11. Iron Transporters (Mitoferrins)

Initially, mitochondrial iron (Fe) transporters, namely Mitoferrins (mIT), were identified and characterized in drosophila, zebrafish and humans [152,153,154]. Plants homologs of mIT were first identified in rice [155] and, recently, two genes encoding for mIT were found in Arabidopsis, and named as *At*mIT1 (At2g30160) and *At*mIT2 (At1g07030) [116]. These proteins have an identity of 81% with each other at the amino acid level and share 38% sequence identity with yeast and 32% identity with zebrafish mIT [116]. In addition, both *At*mIT1 and *At*mIT2 proteins exhibit the classical MCF characteristic feature and were predicted to localize to the mitochondria by proteomic study [156] which has been confirmed by subcellular localization experiments with green fluorescent protein (GFP) fusions and Western blot analyses [116]. The rice mIT protein complemented the growth of yeast mutant which was defective in mitochondrial Fe transport [155]. Similarly, the expression of *At*mIT1 or *At*mIT2 can rescue the phenotype of the yeast mutant defective in mitochondrial Fe transport (mrs3mrs4 mutant; [157]). In mammalian and yeast cells, the redundancy in the roles of mITs has been investigated in terms of biochemical properties and kinetic profiles for Fe^2+^ uptake [154,158]. Moreover, a recent study demonstrated that a purified recombinant mitoferrin^−1^ (TMfrn1), from *Oreochromis niloticus*, catalyzes the transport free Fe and not a chelated Fe complex. In addition, it was shown that it is selective for alkali divalent ions [159]. In the same study, the results indicated that mITs are high-affinity or high-throughput Fe transporters [159]. Of note, mitochondria are known as organelles where there is utilization of other transition metals than Fe, such as manganese, copper, and zinc; however, despite the importance, the mechanisms by which these metal ions are transported through the IMM are not well understood. In addition, it should be mentioned that the possible substrates used by mITs in exchange for the imported Fe are still unknown. In plant systems the biochemical properties of mITs are much less studied than other organisms. Nevertheless, it has been demonstrated that both *At*mIT1 and *At*mIT2 transporters seem to be important for mitochondrial Fe uptake and also for the correct mitochondrial function, and consequently, they are necessary for the proper growth and development of the plant [116,155].

## 5. Conclusions

Research into the metabolic roles of plant MCFs has made impressive advances since the last comprehensive reviews were published some eight to nine years ago [12,135]. This was in part due to be expected, given the massive increase in the number of plant species sequenced in the interim as well as the mechanistic insights into MCF function that were facilitated by recent developments in structural biology. Although, as yet, such experiments have not been carried out for plant proteins, their very high homology to their mammalian counterparts renders the findings based on the human ATP/ADP carrier to likely be highly similar to its plant counterpart and indeed to many other plant MCFs. The genome sequencing has additionally expanded the repertoire of MCFs found in any single species thereby reflecting the challenge that remains in their characterization. That said, as we detail above, via use of heterologous expression, the biochemical characterization of a large number of MCF members has been carried out thereby providing the putative metabolic functions of a substantial number of the family. It is important to state that, as we discuss in the accompanying article [28], experimental proof that these studies do indeed reflect the in vivo role of the proteins remains lacking in some instances. Moreover, a considerable number of MCF proteins remain to be characterized at the biochemical level and such experiments should be a priority for future research. Only once the biochemical potential of each member of the MCF, as well as information concerning their subcellular locations, is acquired alongside that of non-canonical mitochondrial transporters will we be able to accurately model plant mitochondrial function and, for that matter, truly appreciate the importance of this fascinating organelle.

## Figures and Tables

**Table 1:** Mitochondrial carriers (MCs) present in each chromosome of plant genomes recently sequenced.

Dicots	*A. thaliana*		*M. truncatula*		*G. max*		*S. lycopersicum*		*V. vinifera*		*P. persica*		*D. carota*	
*Chr*	mbp	MC N°	mbp	MC N°	mbp	MC N°	mbp	MC N°	mbp	MC N°	mbp	MC N°	mbp	MC N°
1	30	10	46	8	57	8	98	9	23	2	48	12	51	13
2	20	10	56	4	49	11	56	5	19	2	30	8	44	14
3	23	9	57	11	46	7	72	7	19	2	27	8	50	4
4	19	10	44	11	52	10	67	7	24	2	26	2	36	10
5	27	20	35	9	42	7	67	5	25	3	19	8	42	8
6	–	–	49	7	51	9	50	6	22	4	31	12	37	4
7	–	–	46	11	45	10	68	1	21	2	22	3	36	14
8	–	–	37	21	48	16	66	6	22	2	23	7	32	5
9	–	–	–	–	50	5	73	6	23	4	–	–	34	2
10	–	–	–	–	52	4	66	4	18	5	–	–	–	–
11	–	–	–	–	35	3	57	4	20	1	–	–	–	–
12	–	–	–	–	40	2	68	5	23	3	–	–	–	–
13	–	–	–	–	46	7	–	–	24	2	–	–	–	–
14	–	–	–	–	49	5	–	–	30	5	–	–	–	–
15	–	–	–	–	52	4	–	–	20	2	–	–	–	–
16	–	–	–	–	38	7	–	–	22	4	–	–	–	–
17	–	–	–	–	42	6	–	–	17	4	–	–	–	–
18	–	–	–	–	58	6	–	–	29	6	–	–	–	–
19	–	–	–	–	51	8	–	–	24	2	–	–	–	–
20	–	–	–	–	48	6	–	–	–	–	–	–	–	–
Unknown	–	–	28	–	29	–	21	–	59	3	1	–	59	7
Total	119	59	397	82	978	141	828	65	485	60	227	60	421	81
**Monocots**	***B. distachyon***		***S. bicolor***		***Z. mays***		***O. sativa***		***H. vulgare***		***S. italica***		***M. acuminata***	
***Chr***	**mbp**	**MC N°**	**mbp**	**MC N°**	**mbp**	**MC N°**	**mbp**	**MC N°**	**mbp**	**MC N°**	**mbp**	**MC N°**	**mbp**	**MC N°**
1	75	17	81	15	307	14	43	11	558	5	42	7	28	9
2	59	17	78	6	244	8	36	7	768	3	49	5	22	6
3	60	10	74	9	236	7	36	10	700	10	51	7	30	7
4	49	8	69	9	247	10	36	3	647	9	40	4	30	10
5	29	4	72	2	224	13	30	8	67	10	47	11	29	6
6	–	–	61	5	174	5	31	3	583	5	36	3	35	16
7	–	–	66	3	182	4	30	1	657	4	36	5	29	11
8	–	–	63	2	181	12	28	3	–	–	41	3	35	12
9	–	–	59	7	160	9	23	6	–	–	59	18	34	9
10	–	–	61	4	151	5	23	2	–	–	–	–	34	21
11	–	–	–	–	–	–	29	5	–	–	–	–	26	4
12	–	–	–	–	–	–	28	2	–	–	–	–	–	–
Unknown	0	–	25	–	28	3	1	–	249	4	4	–	140	–
Total	271	56	709	62	2134	90	374	61	4229	50	406	63	472	111
	**Algae**				***C. reinhardtii***				***O. tauri***				***M. commoda***	
***Chr***	**mbp**		**MC N°**		**mbp**		**MC N°**		**mbp**		**MC N°**		**mbp**	**MC N°**
1	1		6		1		2		1		5		2	3
2	1		3		1		3		1		3		2	5
3	1		5		1		3		1		5		2	3
4	1		0		0		4		1		0		2	4
5	1		0		0		0		1		0		2	5
6	1		1		1		9		1		1		1	6
7	1		3		1		2		1		3		1	5
8	1		1		1		1		1		1		1	2
9	1		1		1		5		1		1		1	1
10	1		3		1		3		1		3		1	0
11	1		2		0		1		1		3		1	2
12	1		4		1		2		1		3		1	6
13	1		3		1		0		1		2		1	1
14	1		1		0		0		1		1		1	3
15	0		1		0		1		1		1		1	1
16	0		3		1		6		1		2		1	1
17	0		2		1		1		0		3		0	0
18	0		0		–		–		0		2		–	–
19	0		0		–		–		0		0		–	–
20	1		1		–		–		0		0		–	–
21	0		3		–		–		–		–		–	–
Unknown	–		–		10		3		–		–		–	–
Total	13		43		21		46		13		39		21	48

The data were retrieved from comparative genome platform Plaza (https://bioinformatics.psb.ugent.be/plaza/), Ensembl-plants (http://plants.ensembl.org/index.html) and Phytozome (http://www.phytozome.net/). The InterPRO domain used for mitochondrial carrier was ‘IPR023395’. All the sequences were validated by protein blast analysis on the non-redundant database (http://blast.ncbi.nlm.nih.gov/Blast.cgi). The number of MCs refers to sequences which are longer than 265 amino acids and non-redundant. chr, chromosome; mbp, mega base pairs.

**Table 2:** Subfamilies of mitochondrial carrier defined by substrate specificity.

Subfamilies	Aliases	Main Substrates	Triplets *	References
*For nucleotides and dinucleotides*				
ADP/ATP	AAC	ADP, ATP	11 (DNS), 19 (AGT), 23 (KL[G/S]), 84 (TYG), 85 (QRX), 88 (NYV)	[19,80]
Coenzyme A/PAP	CoA/PAP	-	23 (K[V/A]Q), 34 (IVR), 88 ([K/Q]SS)	[65,81]
ATP-Mg/Pi	APC	ATP-Mg, ATP-Ca, Pi, AXP	23 (RQ[Q/A]), 30 (DE[A/T/N]), 84 (EYA), 88 (KDS)	[19,76,82,83,84]
Thiamine pyrophosphate	TPC	Thpp, thmp; (d)NDP, (d)NTP	23 (R[T/S]K), 34 (IT[K/R]), 80 (L[A/T]K), 85 (GAT)	[85,86,87]
Pyrimidine nucleotides	PNC	Pyrimidine (deoxy)nucleotides	19 (G[G/A]K), 27 (CNY), 30 ([D/E]WE), 37 (QQR), 83 ([PEP), 85 (R[I/V][S/T])	[48,88]
FAD/folate	FAD	Folates, FAD	19 (GGK), 27 (HNY), 30 (DWQ)	[70,89,90]
ANT	ANT	ATP, ADP, AMP	19 (SAK), 30 (DAI), 33 (KAK), 37 (QKR)	[46]
NAD^+^	NDT/PXN	NAD+, (d)AMP, (d)GMP	19 (GGK), 27 (CNY), 30 (DWE), 89 (FP[L/F])	[49,68]
GTP/GDP	GGC	GTP, GDP, dgtp, dgdp, ITP, IDP	22 (EGS), 23 (IEL), 84 (QGK), 85 (RSL), 88 (KLS)	[91]
***For di-/tri-carboxylates and keto acids***				
Dicarboxylates	DIC	Malate, succinate, phosphate, sulfate, thiosulfate	26 (TG[C/S]), 27 (H[N/T][S/Q/N]), 33 (K[N/M]K), 88 (RQ[I/L/T])	[42,92,93]
Di-/tri-carboxylates	DTC	Oxoglutarate, citrate	26 (IGS), 27 (QSL), 33 (KLK), 35 (RRQ), 77 (GTY), 84 (YLH), 88 (RMT), 93 ([K/R]DN)	[50]
Citrate/isocitrate	SFC	Citrate, isocitrate, aconitate	22 (EAG), 84 (KNG), 88 (RNT)	[94,95,96]
Citrate	CTP	Citrate, malate, isocitrate, cis-aconitate, PEP	22 (E[A/S][S/T]), 84 (KN[S/D]), 88 (RRV)	[97,98]
2-oxoglutarate	OGC	2-Oxoglutarate, malate	26 (VGS), 27 (QTM), 33 (KLK), 35 (RRR), 77 (GTY), 84 (YVH), 88 (RQT), 93 (TSE)	[43,99]
Oxodicarboxylates	ODC	Oxoadipate, oxoglutarate	22 (EE[A/G]), 77 (PTK), 81 (E[H/N]L) 84 (K[F/W]G), 85 (RNG), 88 (KY[M/L])	[100,101]
Oxaloacetate/sulfate	OAC	Oxaloacetate, sulfate, thiosulfate, a-isopropylmalate	23 (VAA), 26 (TGM), 30 (E[F/Y]D), 80 (YRR), 84 ([L/M]GH), 88 (RQ[C/S])	[47,102]
***For amino acids***				
Glutamate	GC	Glutamate	22 (GQA), 77 (NTR), 80 (LRV), 84 (EFL), 85 (KSF), 88 (KYA)	[103]
Glutamate	BOU	L-Glutamate	-	[104]
Aspartate/glutamate	AGC	Aspartate, glutamate, cysteinesulfinate	22 (GQA), 77 (QCR), 84 (EFQ), 85 (KSF), 88 (KYT)	[45,105]
Aspartate/glutamate	UCP1–2		23 ([D/E][V/I/S/Q][A/V/T/S]), 88 ([R/K] [D/E][F/M])	[12,51]
Ornithine	ORC	Ornithine, (lysine, citrulline, arginine, histidine)	23 ([V/I][A/S]W) but (KSN) in *S. cerevisiae*, 26 (GL[V/C]) but (ELI) in *S. cerevisiae*, 84 (EGA), but (QAV) in atbac2	[106,107,108]
Carnitine	CAC	Carnitine, acylcarnitine	23 (VTW), 85 (FSN)	[44,109]
S-adenosylmethionine	SAMC	S-adenosylmethionine, S-adenosylhomocysteine	19 (G[E/G]G), 23 ([D/E][C/S][A/G]), 26 ([L/F]RT), 80 ([G/A]RW), 85 ([A/S][S/T/D]X), 88 (FQF)	[110,111,112,113]
**For other substrates**				
Phosphate	PiC, mPT	Phosphate	19 (CEG), 23 (HDA), 80 (G[R/K]M), 88 (KKQ)	[114,115]
Iron	MIT, MRS–4, MFRN–2	-	19 (GTG), 22 (E[S/A/H][A/C]), 23 (HDA), 27 ([F/Y][T/N]T)	[12,116]

**Abbreviations:** AAC, ADP/ATP carrier; AGC, aspartate/glutamate carrier; ANT, peroxisomal adenine nucleotide translocator; APC, ATP-Mg/Pi carrier; CAC, carnitine carrier; CoA/PAP, coenzyme A/adenosine 3’,5’-diphosphate carrier; BOU, A bout de soufflé (glutamate transporter); CTP, citrate carrier; DIC, dicarboxylate carrier; DTC, di-/tri-carboxylate carrier; FAD, FAD carrier; GC, glutamate carrier, GGC, GTP/GDP carrier; NDT, NAD^+^ carrier; OAC, oxaloacetate/sulfate carrier; ODC, oxodicarboxylate carrier; OGC, oxoglutarate carrier; ORC, ornithine carrier; PiC, phosphate carrier; mPT, mitochondrial phosphate carrier; PNC, pyrimidine nucleotide carrier; SAMC, S-adenosylmethionine carrier; SFC, succinate/fumarate carrier; TPC, thiamine pyrophosphate carrier; UCP, uncoupling protein. AXP, adenine nucleotides; dNDP, deoxynucleoside diphosphates; dNTP, deoxynucleoside triphosphates; PEP, phosphoenolpyruvate; Pi, phosphate; ThMP, thiamine monophosphate; ThPP, thiamine pyrophosphate. * Symmetry-related amino acid triplets are the triplet sets present in the functionally identified mitochondrial carriers of each family.

## Supplementary Materials

The following are available online at https://www.mdpi.com/2218-273X/10/7/1013/s1, Table S1: Ortologous genes of plant mitochondrial carrier family retrieved from Plaza server.
