# Supplementary material for: Metabolic Roles of Plant Mitochondrial Carriers
Source: Biomolecules. 2020 Jul 8;10(7):1013. doi: 10.3390/biom10071013 (PMC7408384; doi:10.3390/biom10071013)
Supplement: Supplementary file 1 [file biomolecules-10-01013-s001.pdf]

Supplemental Table 1. Ortologous genes of plant mitochondrial carriers familes retrieved from Plaza server (https://bioinformatics.psb.ugent.be/plaza/).

Ortologous Genes				Function
A. thaliana	*S. lycopersicum*	*B. distachyon*	*lucimarinus*	Predicted
At3g08580	Solyc01g087070	BD1G29260	OL10G01660	AAC1
At5g13490	Solyc06g069410	BD3G53520	–	AAC2
At4g28390	Solyc07g053830	–	–	AAC3
	Solyc11g062130			
	Solyc11g062190			
At5g17400	Solyc01g014210	–	–	AAC4
At5g56450	Solyc08g063010	BD2G18347	OL03G05390	Unknown1 (AAC?)
At1g78180	Solyc06g068720	BD1G71800	–	Unknown2 (AAC?)
At5g64970	Solyc04g056260	–	–	Unknown3 (AAC?)
	Solyc05g051560	–	–	Unknown3 (AAC?)
	Soluc04g074130	–	–	Unknown3 (AAC?)
At5g61810		BD1G36570	–	APC1
At5g51050		–	–	APC2
At5g07320		–	–	APC3
At3g51870		BD2G09790	OL10G01620	Unknown4 (CoA/PAP?)
At5g01500	Solyc06g82290	–	–	Unknown5 (CoA/PAP?)
At3g20240	Solyc03g123990	BD3G40497	OL02G00320	Unknown6 (Nt)
–		BD4G34100	OL12G03180	Unknown7 (Nt)
–		BD5G26776	–	Unknown8 (Nt)
At3g05290	Solyc01g009810	BD2G26480	–	Unknown9 (Ant1?)
At5g27520	Solyc01g079760	–	–	Unknown10 (Ant1?)
At4g32400	Solyc06g061090	BD2G34270	OL03G01320	Unknown11 (Nt)
–		BD1G36670	OL12G00350	Unknown12 (Nt)
–		BD3G07500	OL15G01450	Unknown13 (Nt)
At4g01100		BD2G14840	OL03G04280	Unknown14 (CoA/PAP?)
At1g14560	Solyc08g081070	BD2G47880	OL08G02620	Unknown15 (CoA/PAP?)
At4g26180	Solyc08g062860	BD3G08557	–	Unknown16 (CoA/PAP?)
	Solyc12g008770		–	Unknown16 (CoA/PAP?)
–		BD2G41500	–	Unknown17 (CoA/PAP?)
At3g53940		BD2G02880	OL07G00540	Unknown18 (Nt)
At3g55640	Solyc09g011360	BD1G66990	–	Unknown19 (Nt)
At2g37890		–	–	Unknown20 (Nt)
At3g21390	Solyc04g050040	BD2G59930	OL13G02340	Unknown21 (TPC?)
At5g48970	Solyc12g099640	–	OL21G00660	Unknown22 (TPC?)
	Solyc01g067070	–		Unknown22 (TPC?)
At5g66380	Solyc02g088770	BD1G09560	OL13G02140	FAD1 (folate?)
–		–	OL21G00840	FAD2
At2g47490	Solyc09g075490	BD2G28120	OL11G01860	NDT1
At1g25380	Solyc05g009020	–	–	NDT2
At2g39970	Solyc05g052640	BD1G69370	OL20G02530	Unknown23 (NT)
		BD1G67180	–	Unknown24 (NT)
At2g22500		BD3G38410	–	DIC1
At4g24570	Solyc09g031680	BD4G32510	–	DIC2
At5g09470	Solyc05g051400	–	–	DIC3
	Solyc11g010500	–	–	DIC3
–		–	OL14G02490	Unknown25 (DIC?)

At5g19760	Solyc01g005620	BD2G06520	OL10G00520	DTC
	Solyc01g021740			DTC
At4g03115	Solyc08g074370	BD2G32600	–	Unknown26 (DTC?)
At5g01340	Solyc10g085220	BD1G65510	OL07G03670	SFC1
At4g11440		–	–	Unknown27 (acids)
At5g42130	Solyc04g071010	BD2G61677	OL16G01220	Unknown28 (acids)
–	Solyc11g0077070	–	OL03G02490	Unknown29 (acids)
–	Solyc04g071290	–	OL11G02620	Unknown30 (acids)
At2g33820	Solyc02g077910	BD1G15960	OL01G00440	ORNITHINE1
	Solyc10g009090			ORNITHINE1
At1g79900	Solyc03g120100	BD2G07420	–	ORNITHINE2
	Solyc06g066680		–	ORNITHINE2
–	Solyc08g060020	–	OL02G00080	Unknown31 (ornithine)
At5g46800	Solyc06g061170	BD3G34077	–	Unknown32 (amino acid)
At4g27940	Solyc08g082020	BD3G34090	OL12G01640	Unknown33 (amino acid)
At2g46320	Solyc03g007430	BD2G07390	OL01G03790	Unknown34 (amino acid)
–		BD3G34820	–	Unknown35 (amino acid)
At4g39460	Solyc04g051580	BD1G71410	OL01G03770	SAMC1
At1g34065	Solyc12g056600	BD1G01770	–	SAMC2
At1g74240		BD1G70017	OL13G02410	Unknown36 (samc?)
At2g26360		BD4G45190	OL21G00590	Unknown37 (samc?)
At2g35800	Solyc10g079200	–	–	Unknown38 (samc?)
–		BD5G16330	OL17G01690	Unknown39 (amino acid)
At3g54110	Solyc09g011920	BD4G09060	OL07G04120	UCP1
At5g58970		BD2G40390	–	UCP2
At1g14140	Solyc05g007730	BD5G11757	–	UCP3
At2g30160	Solyc01g095570	BD1G65170	OL06G04180	MRS3
	Solyc01g011670			
At1g07030	Solyc01g095510	–	–	Mrs4
At2g17270	Solyc06g072510	BD4G37420	OL17G01410	Unknown40 (phosphate?)
At5g14040	Solyc03g097840	BD3G57890	–	PIC1
At3g48850	Solyc06g072510	BD1G67330	–	PIC2
–		BD4G32020	OL12G02770	Unknown41 (phosphate?)
–		BD5G11740	OL01G01320	Unknown42 (phosphate?)
At5g26200		BD1G72940	OL09G02540	Unknown43
At1g72820	Solyc12g096030	BD1G08301	OL16G02350	Unknown44
	Solyc03g121920			Unknown44
At5g15640	Solyc02g089110	BD4G31560	–	Unknown45
At4g15010		BD2G12390	–	Unknown46
–		BD3G51060	–	Unknown47
